# Preoperative MRI Evaluation of Hand Vessels in Children With Congenital Syndactyly Malformation by a Contrast-Enhanced Three-Dimensional Water-Selective Cartilage Scan

**DOI:** 10.3389/fped.2022.880954

**Published:** 2022-04-06

**Authors:** Bo Liu, Jinhua Cai, Xiaofei Tian, Kaiping Huang, Daisong Liu, Helin Zheng, Longlun Wang, Jing Yang, Hongrong Xu

**Affiliations:** ^1^Department of Radiology, Children's Hospital of Chongqing Medical University, Chongqing, China; ^2^Ministry of Education Key Laboratory of Child Development and Disorders, Children's Hospital of Chongqing Medical University, Chongqing, China; ^3^National Clinical Research Center for Child Health and Disorders, Children's Hospital of Chongqing Medical University, Chongqing, China; ^4^Chongqing Key Laboratory of Pediatrics, Children's Hospital of Chongqing Medical University, Chongqing, China; ^5^Department of Burn and Plastic Surgery, Children's Hospital of Chongqing Medical University, Chongqing, China

**Keywords:** syndactyly, arteries, hand, magnetic resonance imaging, children

## Abstract

**Background:**

It is crucial to preoperatively assess the arteries of the hands in congenital syndactyly malformation (CSM) patients because this information can affect the therapeutic outcome and prognosis.

**Objective:**

To investigate the value of a contrast-enhanced three-dimensional water-selective cartilage scan for the preoperative evaluation of CSM in children.

**Materials and Methods:**

Contrast-enhanced three-dimensional water-selective cartilage scan 3.0 T magnetic resonance imaging (MRI) performed in 16 clinically diagnosed CSM patients with 17 affected hands. The arteries of the hands were displayed with a focus on the bifurcation position of the common palmar digital arteries (CPDAs) and the maturity of the proper palmar digital arteries (PPDAs). The MRI results were interpreted by consensus between two experienced pediatric radiologists with 10 years of MRI experience each. The MRI findings were compared with the operation results.

**Results:**

Of 51 CPDAs in the 17 affected hands, MRI showed that 30 had an abnormal bifurcation position and 20 had a normal position, and of the 102 PPDAs, 14 were shown to have an abnormal maturity and 85 a normal state, which were confirmed by surgery. The accuracy, sensitivity and specificity for determining the bifurcation position of the CPDAs based on MR maximum intensity projection reconstructed images were 98.04% (50/51), 96.77% (30/31) and 100% (20/20), respectively. The maturity of the PPDAs was judged by MR maximum intensity projection reconstructed images with an accuracy, sensitivity and specificity of 97.06% (99/102), 82.35% (14/17) and 100% (85/85), respectively.

**Conclusion:**

Contrast-enhanced three-dimensional water-selective cartilage scan has excellent performance in displaying the bifurcation position of the CPDAs and the maturity of the PPDAs and is of high value for the preoperative evaluation of CSM in children.

## Introduction

Congenital syndactyly malformation (CSM) is one of the most common hand anomalies due to the failure of separation of developing fingers during organogenesis and is characterized by the fusion of adjacent digits (1–3). CSM is usually classified as simple syndactyly, complex syndactyly and complicated syndactyly (4–7). Complicated syndactyly implies the presence of bony abnormalities, such as absent or accessory phalanges within the fused interspaces, which increase the risk of neurovascular abnormalities (1, 8, 9). The separation of syndactyly by operation is the current mainstay therapeutic method (4, 8–11). Before surgery, it is crucial for surgeons to determine the bifurcation of the common palmar digital arteries (CPDAs) and the maturity of the proper palmar digital arteries (PPDAs) of the affected hand, which guarantees a better prognosis and prevention of serious vascular compromise (12–14). Currently, the imaging methods for preoperative assessment of palmar digital vessels include ultrasound, computed tomography angiography (CTA) and magnetic resonance imaging (MRI) (1, 10, 15, 16). However, CTA involves ionizing radiation, and visualization of the soft tissue of hands is not satisfactory. Ultrasound is more operator-dependent (15). MRI involves non-ionizing radiation and provides higher soft tissue resolution than CTA, and its original data allow a three-dimensional reconstruction (17).

Some researchers have used contrast-enhanced magnetic resonance angiography (CE-MRA) to display the bifurcation of the CPDAs and the maturity of the PPDAs in healthy adult hands (18–22). However, to our knowledge, few studies have applied CE-MRA to the preoperative evaluation of children with CSM. Although CE-MRA is often performed using a three-dimensional spoiled gradient echo sequence with fat saturation and the original images can be reconstructed to show the vessels of the hands by maximum intensity projection (19, 23), the phalanges, especially the cartilage of hands, cannot be clearly displayed because their signals are suppressed and removed. Three-dimensional water-selective cartilage scan (3D-WATSc) is another spoiled gradient echo sequence for evaluating cartilage. To date, there have been few reports on the application of 3D-WATSc for the preoperative evaluation of children with CSM. We hypothesized that 3D-WATSc combined with contrast enhancement would clearly display the position of the arteries of hands.

Here, contrast-enhanced 3D-WATSc (CE-3D-WATSc) was performed in a group of pediatric patients with a clinical diagnosis of CSM to display the bifurcation position of the CPDAs and the maturity of the PPDAs. Our aim was to investigate the value of CE-3D-WATSc for the preoperative evaluation of CSM, and we expected to discover that CE-3D-WATSc was a relatively reliable and non-ionizing radiation imaging method for the preoperative assessment of CSM.

## Materials and Methods

### Patients

Sixteen patients with a clinical diagnosis of CSM (11 males, 5 females; mean age, 27.1 ± 8.0 months; age range, 6–123 months) who underwent separation of syndactyly at our institution were included in this study between February 2018 and April 2020. The inclusion criteria were patients diagnosed with CSM by physical examinations (24), radiography (25) and MRI. The exclusion criteria included refusing further treatment, unsuccessful MRI, or follow-up of <1 year. All the patients underwent separation of syndactyly by surgery. The clinical follow-up exceeded 1 year.

### Radiographic Data Collection

For all the patients, radiography of the affected hands was performed using a digital radiography imaging system (Discovery XR650, GE Medical Systems, Milwaukee, WI, USA).

### MRI Acquisition and Analysis

Uncooperative subjects were sedated with 0.5 ml/kg 10% chloral hydrate (made at the Children's Hospital of Chongqing Medical University) administered orally 20 min before MRI examination. MRI was performed with a 3.0-T unit (Achieva, Philips, Eindhoven, The Netherlands) using four surface coils, namely, two SENSE-Flex-S coils and two SENSE-Flex-M coils. Supine and feet-advanced positions were used for all the patients. Images were obtained in the axial, sagittal and coronal planes. The scanning field of view covered from the distal end of the radius and ulna to the tail end of the fingers. In addition, routine T2-weighted (T2WI) and T1-weighted (T1WI) images of the axial, sagittal and coronal planes were obtained with the following parameters: repetition time/echo time, 1572–5400/100 ms for T2WI and 500/20 ms for T1WI; field of view, 16 × 16 cm −20 × 20 cm; slice thickness, 3–5 mm; slice gap, 0.3–0.5 mm; and number of excitations, 2–4. Then, after a body-weight-adapted dose (0.1 mmol/kg) of gadopentetate dimeglumine (Magnevist, Bayer, Berlin, Germany) (23) was manually injected and flushed with the same volume of saline, at the same flow rate when possible, the CE-3D-WATSc sequence was immediately performed with the following parameters: repetition time/echo time, 20/5 ms; slice thickness, 1.5 mm; slice gap, 0.8 mm; field of view, 16 × 16 cm −20 × 20 cm; matrix, 400 × 400; number of excitations, 4; flip angle, 15 degrees; no flow compensation; and fat suppression employed with the principle of selective excitation technique whose pulse type was 1331. The average imaging time of CE-3D-WATSc was ~4 mins. Finally, contrast-enhanced and fat-suppressed T1WI images of the axial, sagittal and coronal planes were obtained, with the same parameters as the abovementioned T1WI.

The original coronal CE-3D-WATSc images were reconstructed with maximum intensity projection on a workstation (EWS, Philips, Eindhoven, the Netherlands). To adjust the different slice thicknesses, the CPDAs and PPDAs of the affected hands were clearly shown, including their bifurcation and maturity. The results of the above procedures were interpreted by consensus between two experienced pediatric radiologists each with 10 years of MRI experience who were blinded to the clinical information.

### Surgical Observation

The patients with CSM underwent separation of syndactyly by surgery with a mean delay of 3 days (delay range, 1–7 days) after MRI examinations. The bifurcation of the CPDAs and maturity of the PPDAs were evaluated by an experienced plastic surgeon during the operation. The normal bifurcation of the CPDAs is located within the metacarpophalangeal joints, and the abnormal bifurcation of the CPDAs is located between the phalanges. Bilateral comparison of the PPDAs, which run along the contiguous sides of the index, middle, ring or little fingers, was performed. When the bilateral PPDAs were obviously different, the thin or absent one was diagnosed as abnormal. When the bilateral PPDAs were approximately the same, they were both diagnosed as normal.

### Statistical Analysis

The CE-3D-WATSc findings and intraoperative observations were compared and analyzed. Statistical analyses were performed using Statistical Product and Service Solutions version 20 (IBM Corporation, Chicago, USA). We used the Pearson chi-square test to analyse the bifurcation of the CPDAs and the maturity of the PPDAs. *P* < 0.05 was considered statistically significant.

## Results

### Radiographic Findings

**Figure 1:**
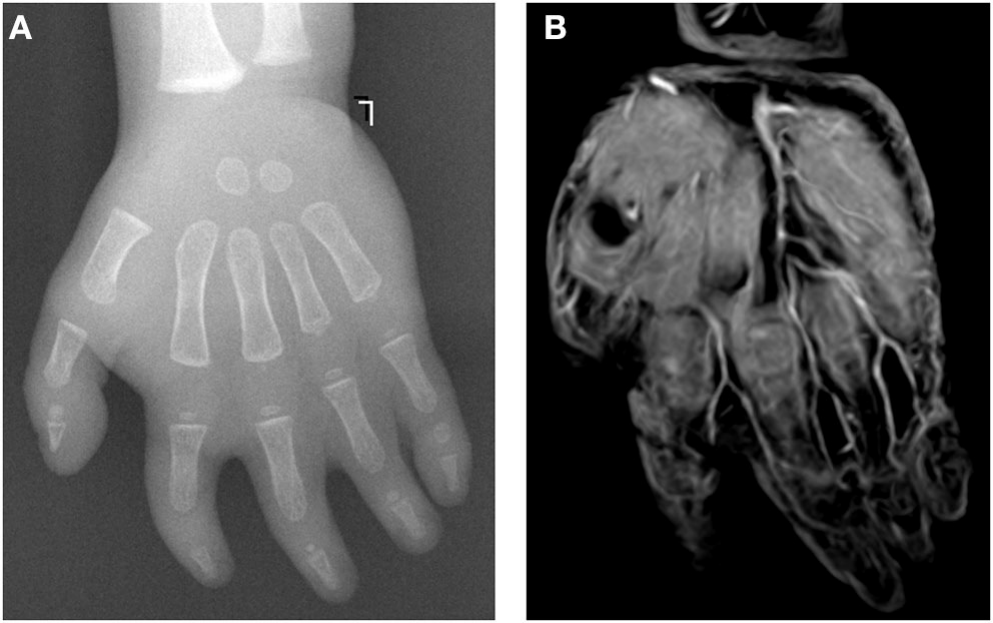
A 15-month-old female patient with complicated syndactyly of the left 2nd-3rd-4th-5th digits. **(A)** The preoperative radiograph showed the fused extent of soft tissues of the 2nd-3rd-4th-5th webspaces and only two phalanges of the 2nd-3rd-4th digits. **(B)** A contrast-enhanced three-dimensional water selective cartilage scan indicated that the bifurcation positions of three common palmar digital arteries of the hand were normal.

**Figure 2:**
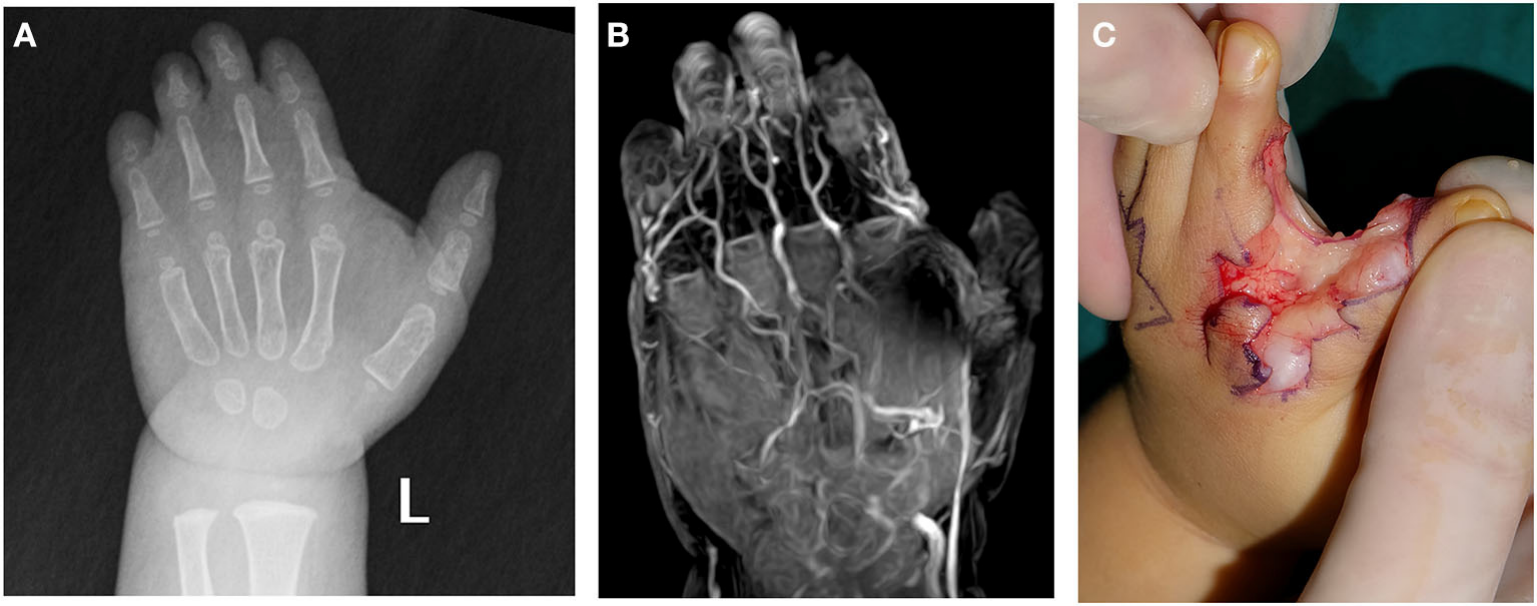
A 17-month-old female patient with complicated syndactyly of the left 2nd-3rd-4th-5th digits. **(A)** The preoperative radiograph showed the fused extent of soft tissues of the 2nd-3rd-4th-5th webspaces and only two phalanges of the 3rd-4th-5th digits. **(B)** A contrast-enhanced three-dimensional water selective cartilage scan indicated that the bifurcation positions of three common palmar digital arteries of the hand shifted distally to approximately one-third of the proximal phalanges. **(C)** These findings were completely consistent with the intraoperative observation results.

**Figure 3:**
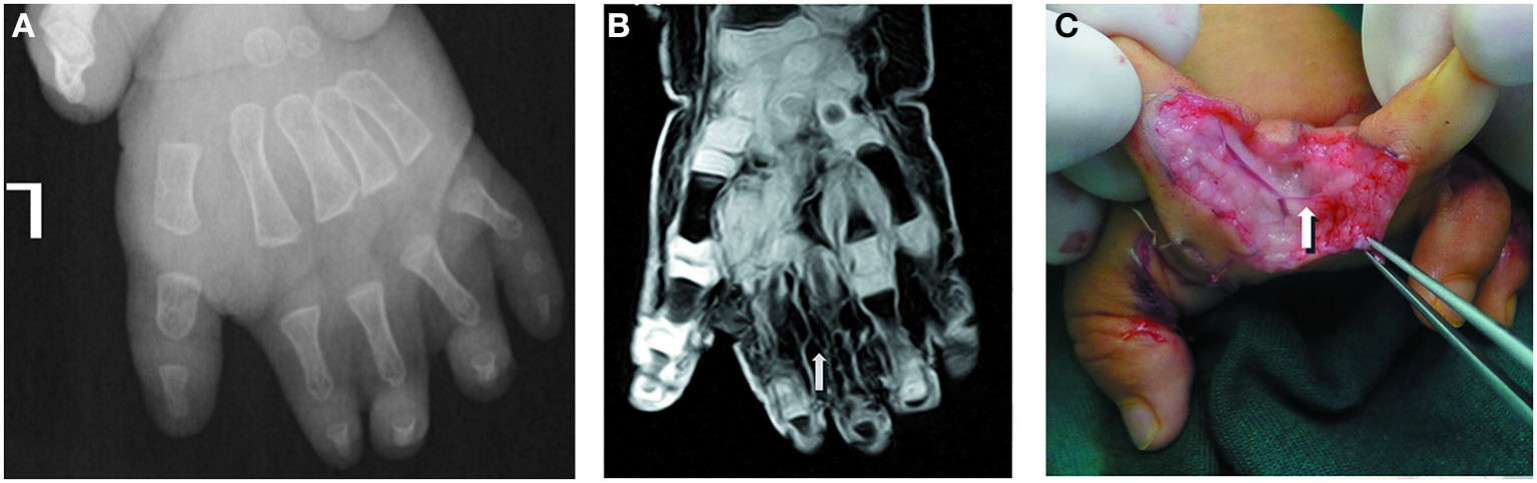
A 13-month-old male patient with complicated syndactyly of the left 1st-2nd-3rd-4th-5th digits. **(A)** The preoperative radiograph showed the fused extent of proximal soft tissues of the 1st-2nd-3rd-4th-5th webspaces and only two phalanges of the 2nd-3rd-4th digits. **(B)** A contrast-enhanced three-dimensional water selective cartilage scan indicated that the proper palmar digital artery of the radial side of the 3rd finger (white arrow) was thin. **(C)** This was completely consistent with the intraoperative observation results.

Sixteen patients were diagnosed with complicated CSM based on the detection of bony abnormalities in phalanges (Table 1); seven of these patients had right syndactyly, eight had left syndactyly, and one had bilateral syndactyly. In the 17 affected hands, 22.35% (19/85) of the fingers were normal, and 77.65% (66/85) were abnormal; additionally, 37.39% (89/238) of the phalanges were dysplastic, namely, 18.91% (45/238) were short and small phalanges, 12.61% (30/238) were absent phalanges (Figures 1A, 2A, 3A), 2.10% (5/238) were thinner phalanges, 1.68% (4/238) were bifurcated phalanges, 1.26% (3/238) were accessory phalanges, and 0.84% (2/238) were thick and big phalanges (Table 1).

**Table 1:** Developmental features of affected hands in patients with complicated CSM based on radiographs (*n* = 17).

	**1st finger**	**2nd finger**	**3rd finger**	**4th finger**	**5th finger**
**Occurrence rate of syndactyly**
Normal	14	1	0	2	2
Abnormal	3	16	17	15	15
**Proximal phalanges**
Normal	17	14	15	16	17
Abnormal	0	3(a^1^+b^1^+c^1^)	2(d^2^)	1(c^1^)	0
**Middle phalanges**
Normal		1	0	2	2
Abnormal		16(a^1^+c^9^+d^1^+e^4^+f^1^)	17(b^1^+c^8^+d^2^+e^6^)	15(c^9^+e^6^)	15(c^9^+e^6^)
**Distal phalanges**
Normal	14	10	16	14	11
Abnormal	3(c^1^+e^2^)	7(a^1^+c^3^+e^2^+f^1^)	1(e^1^)	3 (c^1^+f^2^)	6(c^3^+e^3^)
**Sum of phalanges**
Normal	31	25	31	32	30
Abnormal	3	26	20	19	21

*CSM, congenital syndactyly malformation*.

*a, b, c, d, e and f in the parentheses represent the different types of phalangeal deformity (a, polyphalangism; b, thick and big phalanges; c, short and small phalanges; d, thin and small phalanges; e, hypophalangism; f, bifurcated phalanges). The superscript data of a, b, c, d, e and f in the parentheses represent the number of deformed phalanges*.

### MRI Manifestations

MRI showed that 11 patients had 2nd-3rd-4th-5th-finger syndactyly, three patients had 1st-2nd-3rd-4th-5th-finger syndactyly, one patient had 3rd-4th-finger syndactyly, and one patient had bilateral 3rd-4th-5th-finger syndactyly.

The CPDAs of the 2nd-3rd digits, 3rd-4th digits and 4th-5th digits in the 17 affected hands were all shown by CE-3D-WATSc, of which the bifurcation position was diagnosed as normal in 21 vessels (Figure 1B) and abnormal in 30 vessels. The abnormal bifurcation of the CPDAs was located at approximately one-third of the proximal phalanx in 18 vessels (Figure 2B), at the middle of the proximal phalanx in 7 vessels, and at the distal end of the proximal phalanx in 2 vessels; there was no bifurcation of the CPDAs in 3 vessels (Table 2). Simultaneously, the ulnar PPDA of the 2nd finger, radialis and ulnar PPDAs of the 3rd and 4th fingers and radialis PPDA of the 5th finger were shown by CE-3D-WATSc in 98 vessels and not shown in 4 vessels. On CE-3D-WATSc images, the maturity of the PPDAs was diagnosed as normal in 88 vessels and abnormal in 14 vessels, of which four PPDAs were not shown and ten PPDAs were thin (Figure 3B; Table 3).

**Table 2:** Comparison of the bifurcation position of the CPDAs as shown by CE-3D-WATSc and surgical observation in patients with CSM (*n* = 51).

**Bifurcation position of the CPDAs**	**Of the 2nd-3rd digits**		**Of the 3rd-4th digits**		**Of the 4th-5th digits**		**Sum**	
	**MRI**	**Surgery**	**MRI**	**Surgery**	**MRI**	**Surgery**	**MRI**	**Surgery**
Detected	17	17	17	17	17	17	51	51
Normal	6	6	7	7	8	7	21	20
Abnormal	11 (g^8^+h^1^+j^2^)	11 (g^8^+h^1^+j^2^)	10 (g^4^+h^4^+i^1^+j^1^)	10 (g^4^+h^4^+i^1^+j^1^)	9 (g^6^+h^2^+i^1^)	10 (g^7^+h^2^+i^1^)	30	31

*CE-3D-WATSc, contrast-enhanced three-dimensional water selective cartilage scan; CPDAs, common palmar digital arteries; CSM, congenital syndactyly malformation*.

*g, h, i and j in the parentheses represent the different bifurcation positions of the CPDAs (g, located near the proximal phalanx; h, located in the middle of the proximal phalanx; i, located at the distal end of the proximal phalanx; j, no bifurcation of the CPDAs). The superscript data of g, h, i and j in the parentheses represent the number of different bifurcation positions of the CPDAs*.

**Table 3:** Comparison of the maturity of the PPDAs as shown by CE-3D-WATSc and surgical observation in patients with CSM (*n* = 102).

**Maturity of**	**Ulnar of the**		**Radialis of the**		**Ulnar of the**		**Radialis of the**		**Ulnar of the**		**Radialis of the**		**Sum**	
**the PPDAs**	**2nd finger**		**3rd finger**		**3rd finger**		**4th finger**		**4th finger**		**5th finger**			
	**MRI**	**Surgery**	**MRI**	**Surgery**	**MRI**	**Surgery**	**MRI**	**Surgery**	**MRI**	**Surgery**	**MRI**	**Surgery**	**MRI**	**Surgery**
Detected	16	16	16	15	16	17	16	17	17	17	17	17	98	99
Normal	16	16	15	14	13	13	11	11	16	15	17	16	88	85
Abnormal													14	17
Thin			1	1	3	4	5	6	1	2		1	10	14
Absent	1	1	1	2	1		1						4	3

*CE-3D-WATSc, contrast-enhanced three-dimensional water selective cartilage scan; CSM, congenital syndactyly malformation; PPDAs, proper palmar digital arteries*.

### Surgical Observations

Each CPDA in the 17 affected hands was detected during the operation, and the bifurcation position was normal in 20 vessels and abnormal in 31 vessels. The bifurcation position of the CPDAs shifted distally to one-third of the proximal phalanx in 19 vessels (Figure 2C), at the middle of the proximal phalanx in 7 vessels, and at the distal end of the proximal phalanx in 2 vessels; there was no bifurcation of the CPDA in 3 vessels (Table 2). The maturity of the PPDAs was normal in 85 vessels and abnormal in 17 vessels, of which 3 PPDAs were absent, and 14 were thin to various degrees due to poor development (Figure 3C; Table 3).

There were no significant differences in the bifurcation position abnormity of the CPDAs and dysplasia of the PPDAs between the 2nd, 3rd, 4th and 5th fingers in the 17 affected hands (*P* = 0.921, *P* = 0.158, respectively). However, the bifurcation position abnormity of the CPDAs was significantly higher than the dysplasia of the PPDAs [60.78% (31/51) vs. 16.67% (17/102), *P* < 0.001]. Further analyses showed that the bifurcation position abnormity of each CPDA was significantly higher than the dysplasia of its branch vessels (the CPDA of the 2nd-3rd digits vs. the PPDAs of the ulnar 2nd finger and the radialis 3rd finger, *P* < 0.001; the CPDA of the 3rd-4th digits vs. the PPDAs of the ulnar 3rd finger and the radialis 4th finger, *P* = 0.043; and the CPDA of the 4th-5th digits vs. the PPDAs of the ulnar 4th finger and the radialis 5th finger, *P* < 0.001, respectively).

### Comparison of the MRI Manifestations and Surgical Observations

The diagnostic accuracy, sensitivity, specificity, positive predictive value, and negative predictive value for determining the bifurcation position of the CPDAs based on MRI were 98.04% (50/51), 96.77% (30/31), 100% (20/20), 100% (30/30), and 95.24% (20/21), respectively (Table 2). The diagnostic accuracy, sensitivity, specificity, positive predictive value, and negative predictive value for determining the maturity of the PPDAs based on MRI were 97.06% (99/102), 82.35% (14/17), 100% (85/85), 100% (14/14), and 96.59% (85/88), respectively (Table 3).

## Discussion

The surgical protocol for CSM usually includes three key parts: separation of syndactyly, plasty of syndactylous webs, and surface coverage of the wound (4, 15, 26, 27). Different surgical modes are chosen according to the bifurcation site of the hand vessels when the fingerwebs are separated and take shape (28). Previous studies have shown major variations in the size and line of the PPDAs during separation of syndactyly and plasty of syndactylous webs (8, 12). Even when meticulous surgical operations are performed, postoperative vascular crisis of the finger may still take place (12, 29). Therefore, accurate evaluation of the deformed degree of syndactylous vessels should be performed to efficiently reduce the surgical risk and improve the prognosis. In this study, we applied CE-3D-WATSc MRI for vascular assessment of the hands in CSM, and the results showed that this method could display the bifurcation position of the CPDAs and the maturity of the PPDAs with high diagnostic accuracy. The excellent performance of CE-3D-WATSc in displaying CPDAs and PPDAs recommends this as a promising imaging method for the preoperative evaluation of CSM in children.

The CE-3D-WATSc method used in this study has some advantages in displaying the bifurcation position of the CPDAs and the maturity of the PPDAs. First, 1331 principle of selective excitation technique long pulses was used to inhibit fat signaling, and the fat suppression effect of the CE-3D-WATSc sequence was better than that of the conventional T1WI fat-removal enhanced sequence. The principle of this selective excitation technique is a selective excitation technology. By using frequency-selective and spatially selective excitation pulses (binomial radio frequency pulses) to perform selective water excitation, the fat signal and artifacts caused by fat movement can be eliminated, and the contrast degree of the images can be improved without adding the scanning time. Second, CE-3D-WATSc is a volumetric scanning sequence of T1WI that employs a small thickness of 1.5 mm, a slice gap of 0.8 mm, and a matrix of 400 × 400. The original images can be used for both tracking and reformating the hand vessels to accurately display the bifurcation position of the CPDAs and the maturity of the PPDAs. In addition, the 3D-WATSc sequence was originally designed for displaying cartilage, which is especially suitable for children whose hand bones are developing. We applied this sequence for contrast-enhanced imaging to clearly display the hand vessels and cartilage. The results showed that the clearly displayed phanlanx cartilage provided a definite reference for determining the position of the CPDA vasculature and its bifurcation.

Although the performance of CE-3D-WATSc for displaying the bifurcation position of the CPDAs and the maturity of the PPDAs was outstanding in general, there was still some discrepancy between the imaging findings and the surgical observations for a few hand vessels, mainly PPDAs. In this case series, one CPDA bifurcation located at approximately one-third of the proximal phalanx and 3 absent or thin PPDAs were misdiagnosed as normal. By reviewing the original images, the bifurcation of the CPDA was positioned inaccurately, possibly due to the influence of different view angles in image reconstruction, and the misdiagnosis of the 3 absent or thin PPDAs could be attributed to the artifacts caused in the image reconstruction process. This suggested that we cannot rely on the reconstructed images alone when displaying the arteries. The original images are also important for tracking hand arteries and should be observed carefully in conjunction with the reconstructed images. In addition, when the images are reformatted, meticulous attention should be given to reduce any artifacts, and the reconstructed images should be adjusted from a multiangle view.

Previous studies have reported that neurovascular dysplasia often occurs in complicated CSM (4, 8, 26, 28). The abnormal bifurcation of the CPDAs in this study was significantly higher than that of the simple CSM reported in previous studies in which the bifurcation position of the CPDAs was mostly normal (4, 8, 26, 28). Notably, although the incidence of the CPDAs with an abnormal bifurcation position was high, dysplasia of the branch vessels was relatively rare. That is, the abnormal bifurcation position does not necessarily affect the development of distal vessels. This knowledge is very important for surgeons when deciding how to treat blood vessels during surgery (8, 30), such as considering severing one PPDA of the finger.

In this study, we also applied radiography examination to show the hand bone development in the CSM patients (31). Skeletal deformities, especially in the middle phalanx, were found to be common (30, 32). The location, type and extent of all abnormal phalanges were clearly demonstrated (31). Considering the advantages of radiographs for displaying hand bones and CE-3D-WATSc for assessing hand vessels, we suggest a combination of these two imaging methods for the preoperative assessment of CSM. Radiography, a convenient and quick imaging method, can be used as a primary examination to show bone abnormalities (31), and CE-3D-WATSc can be performed to assess the hand vessels, especially the position of the CPDAs and the maturity of the PPDAs. These imaging assessments can help surgeons develop a preoperative plan and achieve a good prognosis.

There are some limitations in this study. First, the sample size was relatively small, and the included cases were all complicated syndactyly. Some rare types of vascular malformations may not have been included in the case series. In the future, we will apply CE-3D-WATSc MRI to evaluate more complex hand anomaly corrections, such as type IV hypoplastic thumb, severe radial club hand, atypical symbrachidactyly and Apert Syndrome. Second, we did not compare CE-3D-WATSc with other imaging methods, such as CTA and digital subtraction angiography. This is mainly due to the consideration of radiation safety, long examination time and economic burden. Third, the maturity of the PPDAs was only qualitatively assessed by experienced radiologists and surgeons to determine the normal and abnormal groups; thus, the diameter of the abnormal vessels was not measured. In further studies, the artery size displayed on imaging and during operation should be quantitatively assessed and compared to improve the evaluation accuracy of CE-3D-WATSc for determining PPDAs. In addition, we did not provide the information regarding the relationship between vascular identification and the need of skin grafts. The need for skin grafts is decided on the degree of skin tightness in syndactyly. If the skin in syndactyly is tight, a skin graft can be performed. In contrast, a skin graft is unnecessary if the skin in syndactyly is loose. Before a skin graft is performed, the surgeon should understand the bifurcation position of the CPDAs and the maturity of the PPDAs, especially when the PPDAs have to be mutilated during the plasty of the syndactylous webs. In the future, we will verify the crucial relationship between more accurately identifying the vascular pattern and the need for a skin graft.

In conclusion, we used CE-3D-WATSc to evaluate the vasculature of the hand in children with syndactyly. Compared with regular CE-MRA, CE-3D-WATSc shows both blood vessels and cartilage, which is beneficial for determining the positioning of the CPDAs and their bifurcation. CE-3D-WATSc MRI has a high accuracy in displaying the bifurcation position of the CPDAs and the maturity of the PPDAs, which is critical for making a surgical plan. This MRI method can be used in combination with radiographs for the preoperative evaluation of CSM in children.

## Data Availability Statement

The raw data supporting the conclusions of this article will be made available by the authors, without undue reservation.

## Ethics Statement

The studies involving human participants were reviewed and approved by Institutional Review Board of Children's Hospital of Chongqing Medical University. Written informed consent to participate in this study was provided by the participants' legal guardian/next of kin. Written informed consent was obtained from the individual(s), and minor(s)' legal guardian/next of kin, for the publication of any potentially identifiable images or data included in this article.

## Author Contributions

BL: conceptualization, funding acquisition, investigation, methodology, and writing—original draft. JC: supervision and writing—review and editing. XT: validation. KH: data curation. DL: project administration. HZ: visualization. LW: software. JY: resources. HX: formal analysis. All authors contributed to the article and approved the submitted version.

## Funding

This study was supported with funding from the Chongqing Science and Technology Commission (No. cstc2019jscx-msxmX0162).

## Conflict of Interest

The authors declare that the research was conducted in the absence of any commercial or financial relationships that could be construed as a potential conflict of interest.

## Publisher's Note

All claims expressed in this article are solely those of the authors and do not necessarily represent those of their affiliated organizations, or those of the publisher, the editors and the reviewers. Any product that may be evaluated in this article, or claim that may be made by its manufacturer, is not guaranteed or endorsed by the publisher.

## Abbreviations

CSM: congenital syndactyly malformation
CPDAs: common palmar digital arteries
PPDAs: proper palmar digital arteries
CTA: computed tomography angiography
MRI: magnetic resonance imaging
CE-MRA: contrast-enhanced magnetic resonance angiography
CE-3D-WATSc: contrast-enhanced three-dimensional water-selective cartilage scan
T1WI: T1-weighted.
